# Modifications of Phase Morphology, Physical Properties, and Burning Anti-Dripping Performance of Compatibilized Poly(butylene succinate)/High-Density Polyethylene Blend by Adding Nanofillers

**DOI:** 10.3390/polym15224393

**Published:** 2023-11-13

**Authors:** Kartik Behera, Chien-Hsing Tsai, Yen-Hsiang Chang, Fang-Chyou Chiu

**Affiliations:** 1 Department of Chemical and Materials Engineering, Chang Gung University, Taoyuan 333, Taiwan; b.kartik1991@gmail.com (K.B.); a0970600013@gmail.com (C.-H.T.); 2 Department of General Dentistry, Chang Gung Memorial Hospital, Taoyuan 333, Taiwan; chy4d25@cgmh.org.tw

**Keywords:** poly(butylene succinate), high-density polyethylene, blend, nanofillers, anti-dripping, physical properties

## Abstract

A twin-screw extruder was used to fabricate poly(butylene succinate) (PBS)/high-density polyethylene (HDPE) blends (7:3 weight ratio) and blend-based nanocomposites. Carbon nanotubes (CNTs), graphene nanoplatelets (GNPs), and organoclays (15A and 30B) served as the nanofiller, while maleated HDPE (PEgMA) acted as an efficient compatibilizer for the blend. In the composites, individual nanofillers were mostly localized in HDPE domains, but some fillers were also observed at PBS–HDPE interfaces. The sea–island morphology of the compatibilized blend evolved into a pseudo-co-continuous morphology in the composites. Differential scanning calorimetry results confirmed that PEgMA with HDPE evidently accelerated the crystallization of PBS in the blend. The possible nucleation effect of added fillers on PBS crystallization was obscured by the formation of quasi-connected HDPE domains, causing fewer PBS nucleation sites. The presence of nanofillers improved the thermal stability and burning anti-dripping behavior of the parent blend. The anti-dripping efficiency of added fillers followed the sequence CNT > 15A > 30B > GNP. The rigidity of the blend was increased after the formation of nanocomposites. In particular, adding GNP resulted in 19% and 31% increases in the Young’s modulus and flexural modulus, respectively. The development of a pseudo-network structure in the composites was confirmed by measurement of rheological properties. The electrical resistivity of the blend was reduced by more than six orders of magnitude at 3 phr CNT loading, demonstrating the achievement of double percolation morphology.

## 1. Introduction

Polymeric products have gained widespread use in daily life. However, the majority of them are made with a single-component polymer, which may not possess the necessary properties to fully meet the demands of individuals. Therefore, the development of polymeric materials is oriented towards multi-component blends and composites consisting of different polymers and fillers, which have better properties than individual polymers [1,2]. For instance, the modification of electrical properties can be accomplished by loading conductive fillers into a polymer matrix to form composites [3,4,5]. Nanocomposite-based technology has enhanced the physical properties of various polymers and polymer blends by incorporating suitable nanofillers. In order to achieve high-performance polymer nanocomposites, the polymer matrix and nanofiller must be compatible, and the nanofiller must be effectively dispersed in the matrix. Of the existing nanofillers, carbon nanotubes (CNTs), graphene nanoplatelets (GNPs), and organoclays are intensively investigated because of their exceptional aspect ratio and unique properties for improving the properties of polymer matrices [6,7,8].

Polyethylene (PE) is one of the frequently utilized non-polar polymers. PE is a widely used commodity polymer, known for its good mechanical/thermal properties, processability, and low cost. However, it has some drawbacks such as limited temperature resistance, flammability, low stiffness, and limited barrier properties. The widespread utilization of non-sustainable PE has resulted in severe, long-lasting environmental and waste management issues [9]. The applications of PE have been further broadened by blending with other polymers and/or loading with various fillers [10,11,12]. A realistic solution to mitigate the harmful effects is to produce PE blends with biopolymers such as poly(butylene succinate) PBS. The PBS/PE blend shows an immiscible characteristic and has poor physical properties due to a lack of interaction between PBS and PE. Studies have focused on using PE grafted with maleic anhydride (PEgMA) as a compatibilizer/coupling agent to increase interfacial interaction between PE and its blend counterparts or added fillers. PBS is synthesized from the monomers of succinic acid and 1,4-butanediol. Its fine mechanical/thermal properties and processability make PBS one of the most promising sustainable (biodegradable) aliphatic polyesters [13,14,15]. PBS is widely used in textiles, monofilaments, packaging, and medical products [16,17,18]. However, its relatively high cost, low melt viscosity, limited gas barrier properties, and inadequate toughness hinder its commercial feasibility in further applications [19,20]. These shortcomings are expected to be overcome by fabricating PBS-based blends and (nano)composites. The impact of the polarity of two types of organoclay (I.28E and I.34TCN) on the crystallization behavior of PBS was studied and compared by Teng et al. [21]. The crystallization of PBS was significantly improved during isothermal crystallization in the presence of organoclay, with stronger polarity clay exhibiting a greater effect than clay with weaker polarity. Yuan et al. [22] fabricated PBS/CNT nanocomposites and studied their crystallization kinetics and rheological properties. Good dispersion of CNTs was achieved at a low content, while a high CNT content resulted in poor dispersion and agglomeration. CNTs significantly enhanced the crystallization of PBS, resulting in a faster overall crystallization rate. The composites showed elastic characteristics due to the pseudo-network structure formation of CNTs. Platnieks et al. [23] investigated the physical properties of PBS/GNP composites. The crystallinity of PBS increased at 0.5 and 1.0% GNP loadings, whereas it decreased with higher GNP loadings. The thermal conductivity of PBS increased with increasing GNP loading.

Minkova et al. [24] conducted an investigation on the thermal properties and microhardness of high-density polyethylene (HDPE)/15A nanocomposites. PEgMA, ethylene–acrylic acid copolymer (EAA), and acrylic acid grafted PE (PEAA) acted individually as compatibilizers for composite preparation. Composites that contained PEAA and PEgMA had better microhardness, thermal stability, and flame-retardant properties than composites with EAA incorporated. Aontee et al. [25] studied the influence of PEgMA on the mechanical/thermal characteristics and crystallinity of a PBS/HDPE-30/70 blend. The presence of 8 phr PEgMA significantly decreased the crystallinity of the PBS and HDPE. The addition of 2 phr PEgMA increased the tensile strength and elongation at break of the parent PBS/HDPE blend. Kodjie et al. [26] fabricated HDPE/CNT composites by a solvent casting method. The thermal stability of the composites drastically improved due to the radical scavenging function of the added CNTs. The heterogeneous nucleation effect of CNTs was found to result in an increased crystallization temperature of HDPE. Tarani et al. [27] investigated the effect of GNPs with different diameters of 5, 15, and 25 μm on the crystallization kinetics of HDPE. The inclusion of GNPs to HDPE led to a rise in the crystallization temperature. Specifically, the use of GNP M5 (5 μm), which had a smaller diameter, was found to increase the number of heterogeneous nucleation sites, leading to an acceleration in the crystallization rate. Moraweic et al. [28] fabricated nanocomposites by melt blending low-density polyethylene (LDPE) and organoclay. The compatibility of the LDPE–organoclay was improved through the addition of PEgMA as a coupling agent. Transmission electron microscopy (TEM) analysis results revealed that the organoclay in the nanocomposite was dispersed in an exfoliated state. The crystallization temperature of LDPE in the composites remained unaltered as the clay lacked nucleation capability for LDPE. The composites showed better thermal stability than neat LDPE.

Nanocomposite systems made from multi-component polymer blends have been successfully fabricated and characterized for their potential in cutting-edge applications [3,29,30,31]. There have been studies exploring the versatility of PBS and HDPE blend-based composites. Darshan et al. [5] fabricated PBS/HDPE/CNT nanocomposites using PEgMA as a compatibilizer. PEgMA enhanced the compatibility between the two components, while incorporating CNTs increased the thermal stability of the HDPE component. The Young’s modulus (YM) of the composite with 3 phr CNT inclusion showed a 50% increase compared with neat PBS. Wu et al. [32] fabricated PBS/PLA/nitrogen-doped graphene (NG) composites and studied their morphology and physical properties. The NG was mostly dispersed in the PBS matrix, although a small amount of NG was observed in PLA domains. NG improved the miscibility in a PBS/PLA-70/30 blend, resulting in smaller and finely dispersed PLA domains. The inclusion of NG also improved the thermal stability of a PBS/PLA blend. NG loading of 1 wt.% significantly improved the PBS/PLA blend’s tensile properties and dynamic storage/loss moduli. Polypropylene grafted with maleic anhydride (PPgMA) and CNT/organoclay (15A) were used as a compatibilizer and a nanofiller, respectively, for the successful fabrication of PBS/PP blend-based nanocomposites. CNTs were dispersed in both PP and PBS phases, while 15A was selectively localized in the PBS phase, leading to the formation of a pseudo-co-continuous PP–PBS morphology. Composites with 2.5 phr CNT loading revealed a significantly improved YM of the parent blend [8]. HDPE/PP/maleated rubber (EPDM-MA)/15A nanocomposites were fabricated by the melt blending method. The loading of 15A enhanced the PP crystallization, while the crystallization of HDPE was barely affected. The composites revealed improved thermal stability compared with the parent blend. Composites with 15A loading showed significantly increased tensile and flexural properties [33].

In the literature, only a few studies have been conducted on blend-based nanocomposites loaded with different nanofillers. Blends and nanocomposites were fabricated using a melt mixing method, with PBS as the predominant component (70 wt.%). The selection of ratios for fabricating blends and nanocomposites aimed to achieve a balance between the resulting biodegradability and mechanical properties. The comparison of the effect of adding various individual nanofillers on the modification of the phase morphology and physical properties of immiscible polymer blends merits comprehensive investigation in order to broaden the blends’ applications. The present study evaluated the impact of individual incorporation of CNTs, GNPs, and organoclays on the physical characteristics of a PEgMA-compatibilized PBS/HDPE blend [5]. The phase morphology and dispersion of nanofillers in the composites and the thermal properties, burning anti-dripping performance, mechanical properties, electrical resistivity, and rheological behavior of the resultant blends and nanocomposites were determined and compared. The loading with CNTs showed potential for application of the composites in the anti-static and electromagnetic interference fields.

## 2. Materials and Methods

### 2.1. Materials and Sample Preparation

PBS (Bionolle #1001) with an average molecular weight (M_W_) of 13.2 × 10^4^ g/mol was purchased from Showa Denko K.K. (Tokyo, Japan), and HDPE (Taisox 8050) with M_W_ of 2.8 × 10^4^ g/mol was obtained from Formosa Plastic Corporation, Taiwan, China. Maleic anhydride-grafted HDPE (coded as PEgMA, Fusabond MB100D), purchased from DuPont Co. (Wilmington, DE, USA), was used as a compatibilizer for the blend. Organically modified montmorillonites, named Cloisite^®^ 15A and Cloisite^®^ 30B (coded as 15A and 30B, respectively), were purchased from Southern Clay Products Inc., Gonzales, TX, USA. The organic modifiers used for 15A and 30B were detailed in previous reports [3]. The product 15A possesses lower polarity compared with 30B. Multi-walled CNTs (grade ICT-030), having a diameter of 40–90 nm and carbon purity of >90%, were obtained from Golden Innovation Business Co. Ltd., Taiwan, China. GNPs (grade M-5) with an average lateral dimension of 5 μm were obtained from XG-Sciences, Lansing, MI, USA.

The PBS, HDPE, PEgMA, and nanofillers were first dried in a vacuum oven at 70 °C for 24 h. A co-rotating twin-screw extruder (SHJ-20B, L/D = 40) was used to fabricate all the samples tested, including the neat blend components. The mixing temperature from hopper to die was 170 to 190 °C, and the screw speed was 360 rpm. The extruded and pelletized samples were then molded into various ASTM standard specimens using an injection molding machine (V4-20SP-G, Multiplas Enterprise Co., Ltd., Taoyuan, Taiwan) for further characterization. Table 1 records the designations of the samples.

### 2.2. Characterization

The phase morphology of the blends/composites was observed using scanning electron microscopes (Hitachi S-3000N SEM and Jeol JSM-7500F FESEM, Hitachi High-Technologies Corp., Tokyo, Japan). Prior to observation, the samples underwent cryogenic fracturing in liquid N_2_ and were sputter coated with gold. Transmission electron microscopy (TEM) was used to assess nanofiller dispersion in the blend matrix using a Jeol JEM-1230 microscope at 100 kV. Ultrathin sections of the specimens of about 100 nm were prepared using a cryomicrotome (LEICA ULTRACUTR, Deerfield, IL, USA) fortified with a diamond knife at −130 °C. The crystallization and melting behavior of the samples were investigated using differential scanning calorimetry (DSC Q10, TA Instruments, Milford, MA, USA) under a nitrogen environment at a flow rate of 50 mL/min. After melting at 200 °C for 3 min, the crystallization behavior of the samples was recorded on cooling at a rate of 10 °C/min. Subsequently, the samples were heated to 200 °C at 20 °C/min to assess the melting behavior. Samples were tested for thermal stability using a TGA TA Q50 thermogravimetric analyzer at a heating rate of 10 °C/min from room temperature to 700 °C under a nitrogen environment at balance and sample purge flow rates of 40 mL/min and 60 mL/min, respectively.

An X-ray diffractometer (D2, Billerica, MA, USA) with a monochromatic CuKα (λ = 1.54 Å) source was used for crystal structure assessment at a scan rate of 0.02 degree/s. Behera et al. [3,6] described a burning test to evaluate the anti-dripping behavior of fabricated specimens. Specimens (63.0 × 12.5 × 3.0 mm^3^) were brought into direct contact with the flame of an alcohol lamp for 10 s and then removed from the flame to observe subsequent burning/dripping behavior.

Tensile and flexural moduli of specimens in accordance with ASTM D638 (Type V) and ASTM D790, respectively, were determined using a Gotech testing machine (AI-3000, Taichung, Taiwan). Experiments were carried out with a crosshead speed of 10 mm/min or 1 mm/min at room temperature for tensile and flexural tests, respectively. The reported value for each formulation is an average of at least six independent tests. The rheological properties were studied using an Anton Paar Physica rheometer (MCR 101, Anton Paar GmbH, Graz, Austria) at 170 °C in parallel plate geometry (25 mm in diameter, 10 mm in thickness) at 1% strain amplitude. The selected samples were measured for electrical resistivity using Mitsubishi Chemical Co. (Yamato, Japan) MCP-HT450 and MCP-T700 resistivity meters at room temperature.

## 3. Results and Discussion

### 3.1. Phase Morphology and Selective Localization of Nanofiller(s)

**Figure 1:**
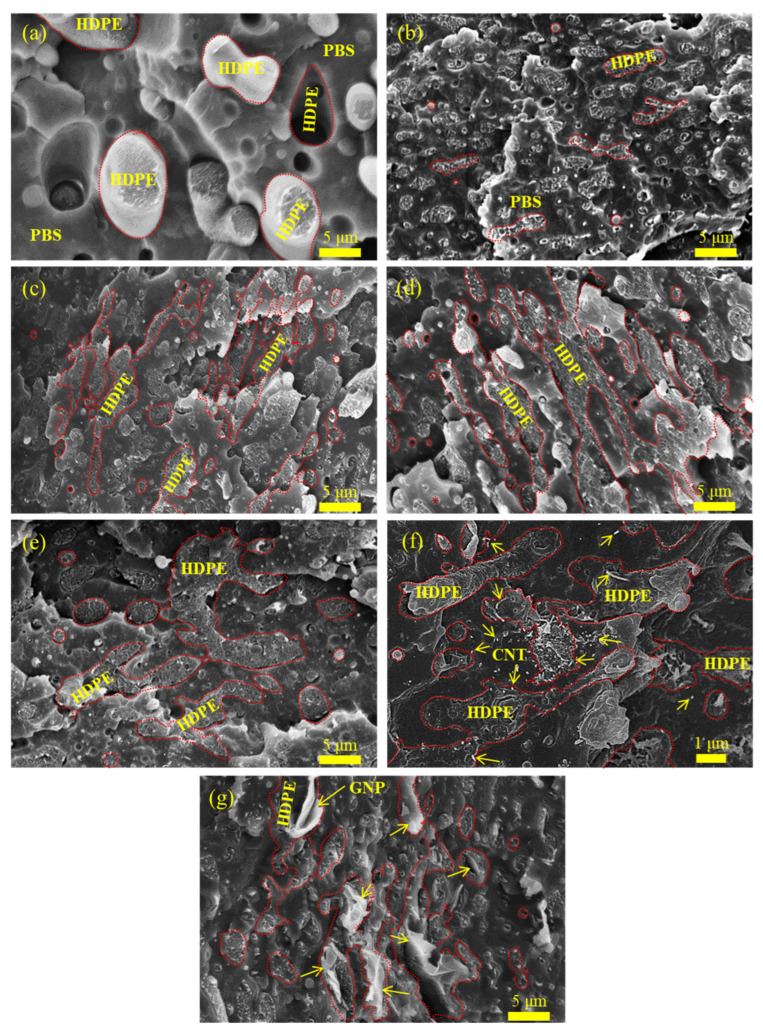
SEM images of representative samples: (**a**) S7E3; (**b**) S7E3M; (**c**) S7E3MA3; (**d**) S7E3MB3; (**e**) S7E3MT3; (**f**) higher magnification SEM image of S7E3MT3, and (**g**) S7E3MG3.

Figure 1 depicts the SEM images of the blends and composites. As shown in Figure 1a, the S7E3 blend displayed a phase-separated (sea–island) morphology, demonstrating its immiscible characteristic. The size of the dispersed HDPE domains (30 wt.% and 36 vol.%) ranged from 2 to 7 µm, with an average diameter larger than 4 µm. No adhesion between the PBS matrix and HDPE domains was observed. The SEM image of PEgMA-added S7E3M is shown in Figure 1b. The pulled-out HDPE domains were less often detected compared with those in S7E3, and the average domain size (some with an elongated shape) was drastically reduced to ca. 2 µm. The loading of PEgMA compatibilized PBS with HDPE through the interaction between the ester linkage of PBS and the MA of PEgMA (miscible with HDPE domains) [5]. Figure 1c,d show the images of S7E3MA3 and S7E3MB3, respectively. A quasi-connected structure of HDPE domains was evident in both composites. However, it was difficult to detect the location of either 15A or 30B in the blend matrix. Figure 1e depicts the image of S7E3MT3, in which the HDPE domains likewise became quasi-connected. As revealed in the higher magnification image (Figure 1f), CNTs were mainly seen in connected HDPE domains due to their non-polar characteristic. A small number of CNTs were also discernible in the PBS phase. Figure 1g depicts the SEM image of S7E3MG3. The larger-sized GNPs (arrowed) were generally located in the HDPE domains and at the interface of PBS–HDPE phases, with some agglomeration. The above modification in the phase-separated morphology of S7E3M after adding individual nanofillers was ascribed to the alteration of the viscosity ratio between the PBS and HDPE phases and the selective localization of the added fillers (see following rheological/TEM results).

**Figure 2:**
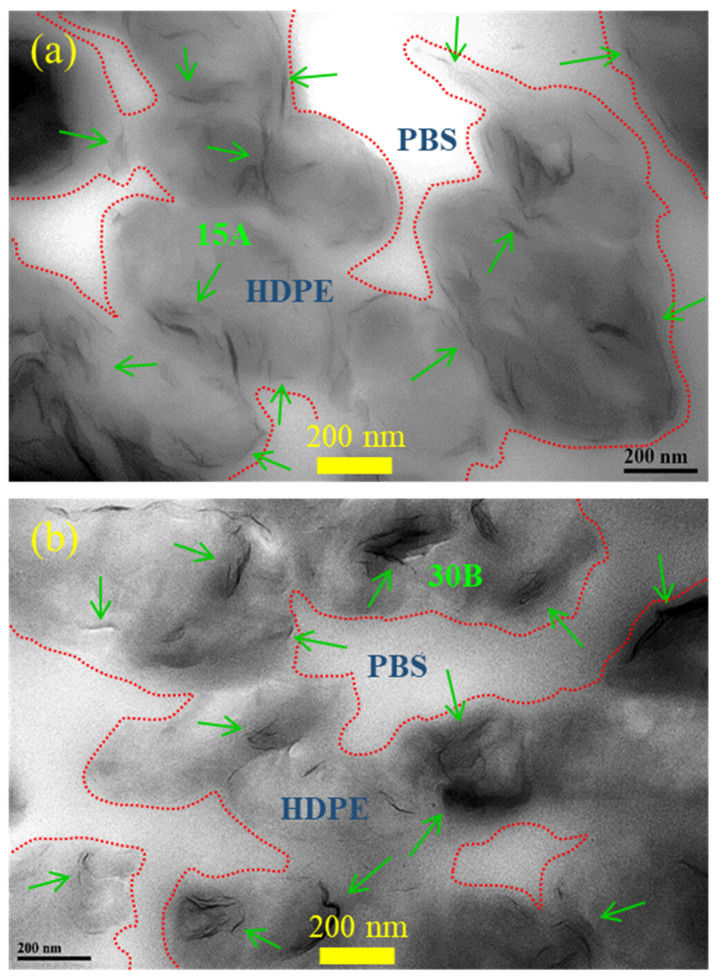
TEM images of selected samples: (**a**) S7E3MA3 and (**b**) S7E3MB3.

The dispersion of 15A and 30B in the composites was inspected by TEM. Figure 2a depicts the image of S7E3MA3, according to which 15A was mainly dispersed in HDPE domains, but some was found at the interface between PBS and HDPE phases. The connected domains of HDPE were observed, consistent with the SEM results. As shown in Figure 2b, 30B was also mostly located in the connected HDPE domains. The dispersion status (some aggregation) of 30B was inferior to that of 15A, which could be due to the better affinity between 15A and the less polar HDPE/PEgMA compared with that of 30B and HDPE/PEgMA.

### 3.2. Crystallization and Melting Behavior

**Figure 3:**
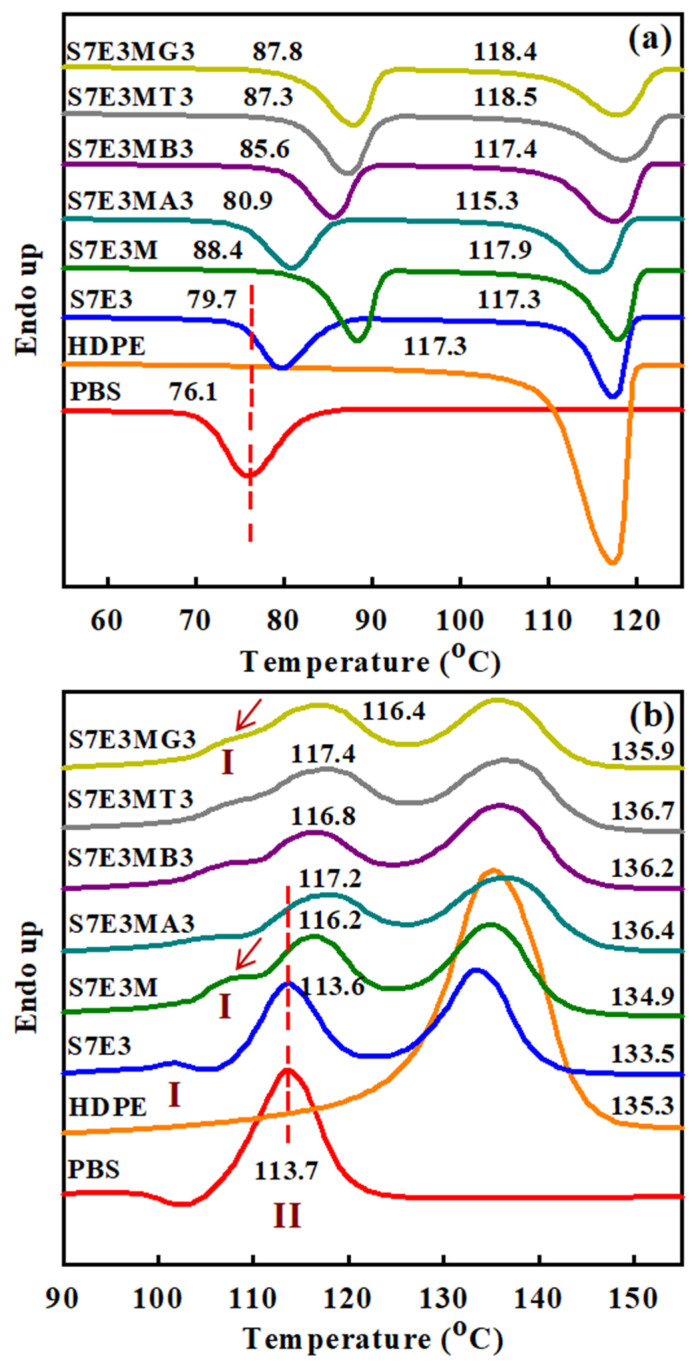
(**a**) DSC curves of the samples at 10 °C/min cooling; (**b**) DSC heating curves of the samples pre-cooled at 10 °C/min.

Figure 3a displays DSC curves of selected samples at 10 °C/min cooling from the melt state. The crystallization peak temperatures (T_p_s) of PBS and HDPE were 76.1 °C and 117.3 °C, respectively. In S7E3, the T_p_ of PBS increased, but T_p_ did not change for HDPE. Hence, the pre-solidified HDPE showed a nucleation effect for PBS crystallization. The presence of PEgMA in S7E3 (cf. S7E3M) resulted in a drastically increased T_p_ of PBS, whereas the T_p_ of HDPE slightly increased. The enhanced affinity (compatibility) of pre-solidified PEgMA/HDPE to PBS and the more dispersed HDPE domains both led to higher nucleation efficiency for PBS crystallization [5]. Regarding the composites, the T_p_ of PBS remained higher than that of neat PBS, and the T_p_ of HDPE was marginally changed due to its intrinsically fast crystallization characteristic. However, the individual incorporation of 3 phr 15A and 30B decreased the T_p_ of PBS compared with that of the parent S7E3M. The retarded crystallization of PBS could be associated with the development of quasi-connected HDPE domains, which caused fewer HDPE/PEgMA phase-induced nucleation sites for PBS crystallization. S7E3MB3 was seen to display a higher PBS T_p_ than that of S7E3MA3, which could be ascribed to a higher affinity of 30B (more polar) to PBS than that of 15A (less polar). Some 30B might be located at the PBS phase to facilitate PBS nucleation. The difference in the phase modification of HDPE domains after adding 15A and 30B individually should also be taken into account for observation. Regarding S7E3MT3 and S7E3MG3, the T_p_ of PBS was higher than that of S7E3MB3, being close to that of S7E3M. CNTs and GNPs, partially localized at the interface of the PBS–HDPE phases, exhibited a nucleation effect for PBS crystallization and thus compensated for the retarded nucleation caused by the development of quasi-connected HDPE domains.

**Figure 4:**
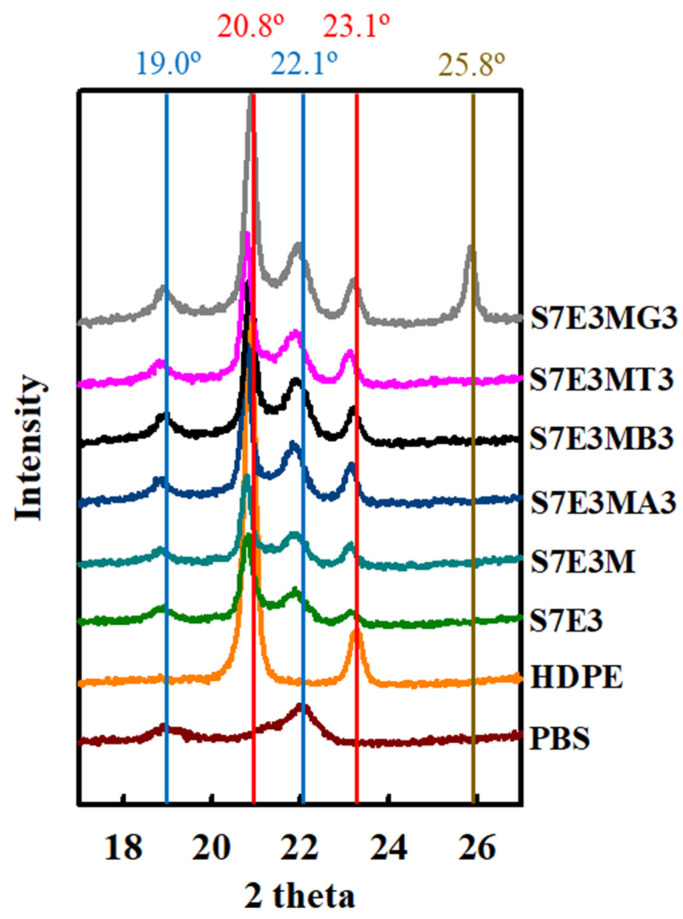
XRD patterns of 10 °C/min-cooled samples.

Figure 3b depicts the melting behavior of 10 °C/min pre-cooled samples heated at 20 °C/min in DSC experiments. A shallow exotherm followed by the main melting peak (melting–recrystallization–remelting) was observed in neat PBS, in which the main melting at approximately 114 °C (T_mII_) was characterized as peak II. Neat HDPE displayed a simple melting at around 135 °C (T_m_). For the S7E3 blend, a minor melting peak I (combined with recrystallization) along with the subsequent main melting peak II of PBS was seen. Compared with neat components, the T_mII_ of PBS hardly changed, and the T_m_ of HDPE slightly decreased in S7E3. Unlike S7E3, S7E3M and the composites showed an evident melting peak I (arrowed, around 107 °C) prior to the main melting peak II of PBS, and the peak II shifted to a higher temperature compared with those of S7E3 and neat PBS. The T_m_ of HDPE was also slightly higher in the composites than in S7E3. The melting behavior change after forming S7E3M, and the composites indicated that the added PEgMA and fillers had induced more stable PBS and HDPE crystal growth. The nucleation effect of PEgMA and added fillers played a role in this observation. Figure 4 shows the XRD results of samples cooled at 10 °C/min from the melt state. Neat PBS showed two diffractions (monoclinic structure) at 2θ = 19.0° (020) and 22.1° (110) [5,17], whereas HDPE revealed two diffractions (orthorhombic structure) at 2θ = 20.8° (110) and 23.1° (200) [5]. Diffractions of both PBS and HDPE crystals were seen in the blends and composites. Additionally, S7E3MG3 showed a strong diffraction peak at 2θ = 25.8°, attributed to the layered structure of GNPs. The XRD results confirmed that the formations of blends and composites did not alter the crystal structures of PBS and HDPE.

### 3.3. Thermal Stability

**Figure 5:**
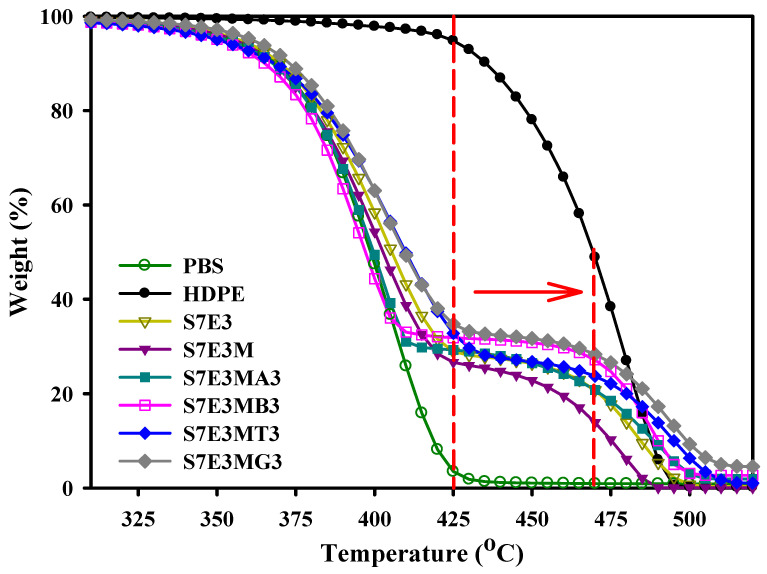
TGA curves of the samples scanned under a N_2_ environment.

TGA curves of selected samples are shown in Figure 5. HDPE showed higher thermal stability than PBS, with a temperature at 10 wt.% loss (T_d10_) of around 435 °C for HDPE and 369 °C for PBS. After forming the blends and composites, two-step degradations were seen, corresponding to the individual degradation of PBS and HDPE. Comparing the T_d10_ of individual samples (Table 2), it could be concluded that the development of quasi-connected HDPE domains in the composites hardly retarded the initial degradation of PBS matrix (similar T_d10_), except for the added GNP showing some enhancing effect. A dotted vertical line was drawn in the figure at 425 °C to indicate the start point for serious degradation of neat HDPE. It can be observed that the start point for serious HDPE degradation evidently shifted to higher temperatures in the composites, particularly for CNT- and GNP-included composites. This result was attributed to the fillers with outstanding thermal stability being mostly located in the HDPE domains. Moreover, CNTs and GNPs could supply additional free radical scavenging functionality [3,6]. The T_d90_ (90 wt.% loss temperature) of the composites followed the sequence of added fillers: GNP > CNT > 30B ≒ 15A. The layered nature of GNPs retarded the evaporation of degraded molecules to a greater degree, thus showing superior enhancement of thermal stability of the composites compared with CNTs.

### 3.4. Anti-Dripping Performance

**Figure 6:**
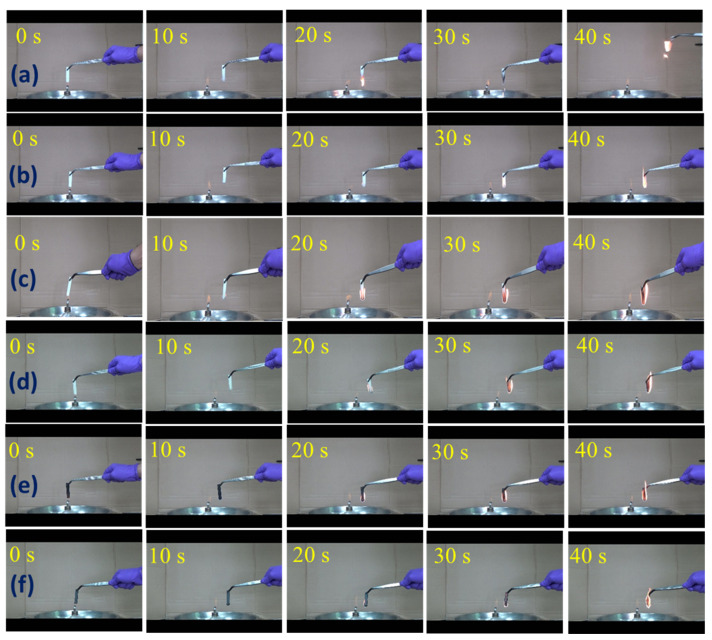
Digital photos of combustion process at different times: (**a**) S7E3, (**b**) S7E3M, (**c**) S7E3MA3, (**d**) S7E3MB3, (**e**) S7E3MT3, and (**f**) S7E3MG3.

The effects of adding PEgMA and nanofillers on the anti-dripping performance for the S7E3 blend are shown in Figure 6 as digital photos of selected specimens at various time intervals. S7E3 (Figure 6a) kept burning after being removed from the flame source and then showed melt dripping after 13 s, leading to viscous flow-like behavior. S7E3 showed severe melt dripping (more than 40 droplets) at up to 40 s of burning, indicating poor melt dripping performance. The compatibilized S7E3M displayed melt dripping at a delayed time of 31 s (Figure 6b), and the flame area/size was smaller compared with S7E3 at the identical time. The improved anti-dripping behavior of S7E3M could result from it having a better dispersion of HDPE domains. S7E3M showed only four melt droplets after 40 s of burning. Figure 6c,d display the burning performance of S7E3MA3 and S7E3MB3, respectively. After continuous burning for 40 s, no melt droplets were observed for both S7E3MA3 and S7E3MB3 composites, indicating that the presence of 15A and 30B improved the anti-dripping properties of the S7E3M blend. Similarly, S7E3MT3 (Figure 6e) showed no melt droplets after 40 s of burning. The flame size of S7E3MT3 was smaller than that of S7E3M [3]. Regarding S7E3MG3 (Figure 6f), it displayed an inferior anti-dripping performance compared with the other composites. Melt droplets were noticed after 48 s of burning, which could be attributed to the worse dispersion of GNPs in the S7E3M matrix (mainly in HDPE domains). The improved anti-dripping performance observed for the composites resulted from the formation of quasi connected filler-included HDPE domains having a higher melt strength than the PBS matrix. The burning test process (see Supplementary Videos S1–S6) showed that S7E3MT3 did not reveal any dripping after 80 s, whereas S7E3MA3, S7E3MB3, and S7E3MG3 showed dripping after 75 s, 57 s, and 48 s, respectively. Hence, it was demonstrated that S7E3MT3 possessed an improved anti-dripping performance compared with S7E3MA3, S7E3MB3, and S7E3MG3. The improvement of anti-dripping performance by adding fillers for S7E3M followed the sequence of CNT > 15A > 30B > GNP.

### 3.5. Mechanical Properties

**Figure 7:**
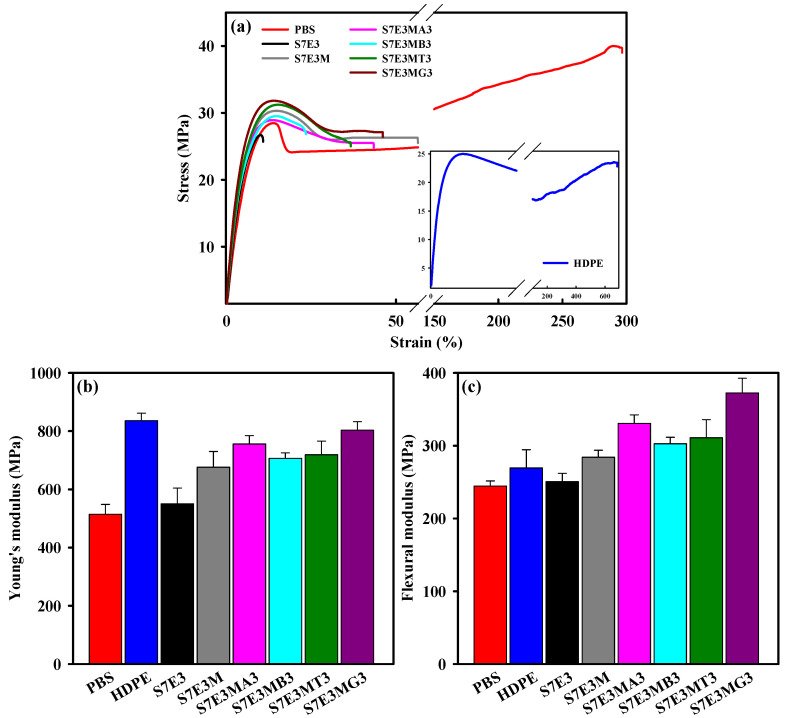
(**a**) Typical S–S curves, (**b**) Young’s modulus, and (**c**) flexural modulus of the samples.

Figure 7a depicts typical stress–strain curves of selected samples. The Young’s modulus (YM) and flexural modulus (FM) of the samples are compared in Figure 7b and 7c, respectively. The data are listed in Table 2. Neat HDPE had a higher YM than neat PBS. The YM of S7E3 was in between the two neat components and was lower than the value (611) calculated by the law of additivity (attributed to the blend’s evidently incompatible characteristic). After incorporating PEgMA, the YM increased to a value higher than 611, demonstrating the enhancement in PBS–HDPE interaction. After loading with different fillers (15A/30B/CNT/GNP), the YM further increased. S7E3MA3 showed a higher YM than S7E3MB3, possibly due to the better dispersion of 15A compared with 30B. Of the fillers added, GNPs showed the best efficiency (up to 19%) in enhancing the YM of S7E3M matrix. The intrinsic high modulus and the sheet-like structure of GNPs played dominant roles in its achievement of the best YM improvement. Similar to the formulation-dependent trend of YM, neat PBS had an FM value lower than that of neat HDPE. The FM of S7E3 was in between those of the parent components. After adding PEgMA, the FM increased (even higher than neat HDPE) because of the compatibilizing effect of PEgMA. Loading of 3 phr of 15A, 30B, CNTs, and GNPs into the S7E3M matrix increased the FM by 16%, 7%, 10%, and 31%, respectively. The added fillers’ efficiency in enhancing the YM and FM followed the sequence of GNP > 15A > CNT > 30B. The reasons behind this significant improvement in FM after adding GNPs are identical to those for YM improvement.

### 3.6. Melt Rheology

**Figure 8:**
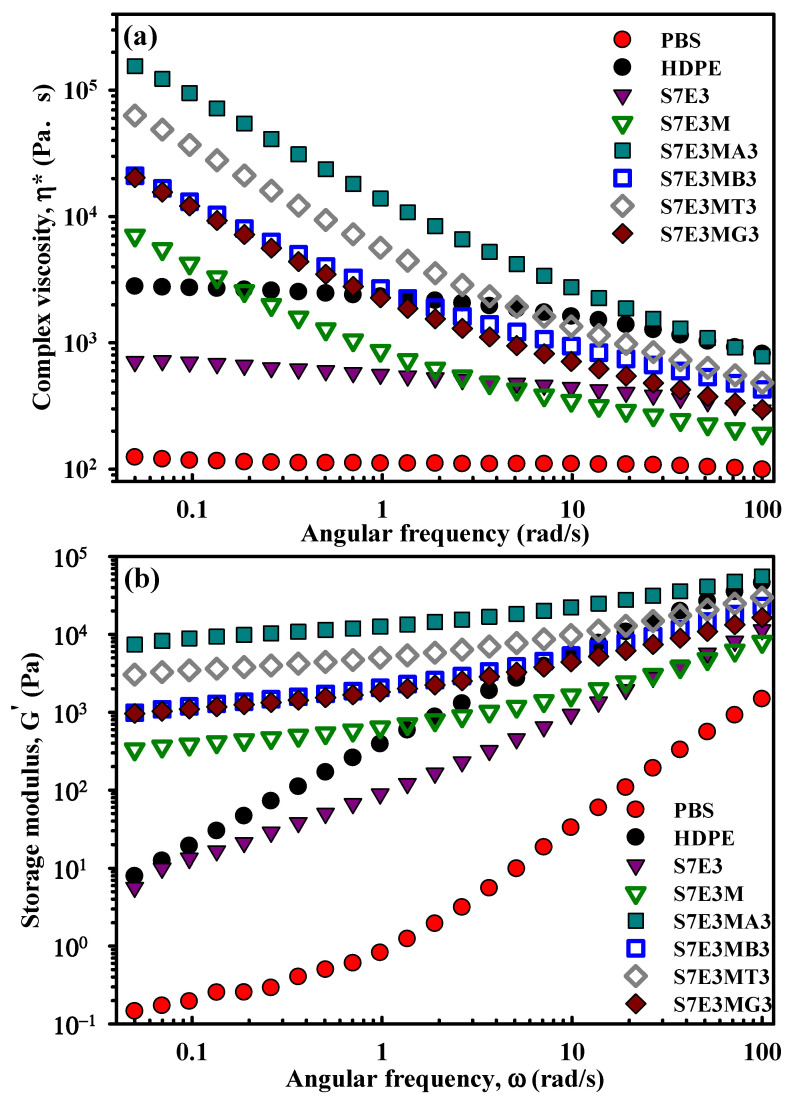
Rheological properties of the samples: (**a**) η* vs. ω and (**b**) G′ vs. ω.

Rheological properties play a vital role in polymer processing and can reveal the internal structure modification after forming blends/composites. Figure 8a depicts the complex viscosity (η*) vs. sweep frequency (ω) of the samples. Neat PBS displayed the lowest viscosity and liquid-like (Newtonian fluid) behavior at the test temperature of 170 °C. Neat HDPE had higher η* values than PBS at all frequencies; S7E3 had η* values that lay between those of PBS and HDPE. With the incorporation of PEgMA, S7E3M showed higher η* values than those of S7E3 at ω < 3 rad/s and revealed shear thinning behavior at all frequencies. The behavior of S7E3M could be ascribed to the more dispersed/reduced domain size of HDPE and the improvement of interaction between PBS and HDPE phases. The η* values of S7E3M increased after forming the composites. The added inorganic fillers reduced the fluidity of the polymer matrix under shear tests. Among the composites, S7E3MA3 displayed the highest η* at all frequencies due to the fine dispersion of 15A along with the good interaction between 15A and the polymer matrix (mainly HDPE/PEgMA domains) [28]. The impact of incorporating individual fillers on the η* of S7E3M matrix followed the sequence 15A > CNT > 30B > GNP. The inferior dispersibility of GNPs in the composite led to its having the smallest effect on η* modification. Moreover, the η* showed non-Newtonian fluid (shear thinning) behavior at the frequencies tested for all composites. The shear thinning behavior at low frequencies confirmed the formation of a pseudo-network (solid-like) structure of quasi-connected HDPE domains combined with fine dispersion of nanofillers. The storage modulus (G′) is plotted against ω in Figure 8b. The G′ increased with increasing ω for the samples tested, and the values were higher for the composites compared with the S7E3M parent matrix. A similar formulation-dependent trend to that of η* was observed in the G′ test results. The G′ of S7E3 was in between that of neat PBS and HDPE. S7E3MA3 displayed the highest G′ of the composites at all frequencies, being about 1.5 orders of magnitude (at ω < 1 rad/s) higher than that of S7E3M. The almost flattened slopes in the plots of S7E3M and composites at low frequencies, agreeing with the η* results, signified the development of a pseudo-network structure (solid-like behavior) within the polymer matrix [32]. Compared with the neat components and blends, the increased elastic nature of the composites was confirmed.

### 3.7. Electrical Resistivity

The measured electrical resistivity (ER) values of the samples are included in Table 2. As anticipated, neat components and the blends had ER values of above 1014 Ω-cm. After adding 15A or 30B into S7E3M (i.e., S7E3MA3 and S7E3MB3), the ER hardly changed, remaining electrically insulating. Notably, the ER value decreased to around 108 Ω-cm for S7E3MT3, more than six orders lower compared with the parent S7E3M. The fine dispersion of CNTs, mainly in the quasi-connected HDPE domains, was responsible for the evident ER drop, suggesting that a double percolation morphology was achieved in S7E3MT3. Nevertheless, with 3 phr GNP loading in S7E3M (S7E3MG3), the ER barely decreased due to the inferior dispersibility of multi-layered GNPs in quasi-connected HDPE domains.

## 4. Conclusions

Immiscible PBS/HDPE blends and blend-based nanocomposites compatibilized with PEgMA were successfully fabricated by melt extrusion. In the composites, the added nanofillers (15A, 30B, CNTs, and GNPs) were mainly localized in HDPE domains, but some were also situated at the PBS–HDPE interfaces, leading to quasi-connected HDPE domains. With the incorporation of PEgMA, crystallization of PBS in the blend was evidently accelerated during the cooling process. The added fillers did not facilitate, and even retarded, the crystallization of PBS, attributed to the combined effects of filler localization mainly in HDPE domains and the formation of quasi-connected HDPE domains (fewer nucleation sites for PBS crystallization). The crystallization of HDPE barely changed in the blends/composites due to its intrinsic fast crystallization characteristic. PEgMA and fillers induced more stable PBS crystals to grow in the compatibilized blend and composites. The composites showed higher thermal stability compared with the parent blend, particularly in enhancement for the HDPE portion. An improvement in anti-dripping performance in burning tests was found for the composites compared with the blends, with the improvement efficiency of the added fillers following the sequence of CNT > 15A > 30B > GNP. The rigidity of the blend improved after the development of composites; the effectiveness of the added fillers in increasing rigidity followed the sequence of GNP > 15A > CNT > 30B. The rheological properties suggested the formation of a pseudo-network structure in the composites. The electrical resistivity of the blend matrix decreased by more than six orders of magnitude to around 108 Ω-cm at 3 phr CNT loading.

## Figures and Tables

**Table 1:** Samples’ designations and formulations.

Sample Code	Composition	Parts (wt.%)
PBS	PBS	100
HDPE	HDPE	100
S7E3	PBS/HDPE	70/30
S7E3M	PBS/HDPE/PEgMA	70/25/5
S7E3MA3	PBS/HDPE/PEgMA/15A	70/25/5/3 *
S7E3MB3	PBS/HDPE/PEgMA/30B	70/25/5/3 *
S7E3MT3	PBS/HDPE/PEgMA/CNT	70/25/5/3 *
S7E3MG3	PBS/HDPE/PEgMA/GNP	70/25/5/3 *

* Parts per hundred polymer resins (phr).

**Table 2:** Thermal, mechanical, and electrical property data of fabricated samples.

Samples	Properties				
	T_d10_ (°C)	T_d90_ (°C)	YM (MPa) (σ)	FM (MPa) (σ)	Log (Volume Resistivity) (Ω-cm)
PBS	369	419	515 ± 34	245 ± 7	>14
HDPE	435	488	836 ± 25	269 ± 25	>14
S7E3	370	484	551 ± 54	251 ± 12	>14
S7E3M	368	475	676 ± 54	284 ± 10	>14
S7E3MA3	368	489	756 ± 29	331 ± 12	>14
S7E3MB3	367	490	707 ± 19	303 ± 9	>14
S7E3MT3	368	495	719 ± 47	311 ± 25	7.5 ± 0.1
S7E3MG3	373	499	803 ± 29	373 ± 20	>14

σ: standard deviation.

## Data Availability

The data presented in this study are available on request from the corresponding author.

## Supplementary Materials

The following supporting information can be downloaded at: https://www.mdpi.com/article/10.3390/polym15224393/s1, Video S1: S7E3.mp4; Video S2: S7E3M.mp4; Video S3: S7E3MA3.mp4; Video S4: S7E3MB3.mp4; Video S5: S7E3MG3.mp4; Video S6: S7E3MT3.mp4.
